# Precipitation shapes communities of arbuscular mycorrhizal fungi in Tibetan alpine steppe

**DOI:** 10.1038/srep23488

**Published:** 2016-03-22

**Authors:** Jing Zhang, Fang Wang, Rongxiao Che, Ping Wang, Hanke Liu, Baoming Ji, Xiaoyong Cui

**Affiliations:** 1 College of Life Sciences, University of Chinese Academy of Sciences, No. 19A, Yuquan Road, Shijingshan District, Beijing, 100049, China; 2 College of Forestry, Beijing Forestry University, No. 35, Tsinghua East Road, Haidian District, Beijing, 100083, China

## Abstract

Tibetan Plateau is one of the largest and most unique habitats for organisms including arbuscular mycorrhizal fungi (AMF). However, it remains unclear how AMF communities respond to key environmental changes in this harsh environment. To test if precipitation could be a driving force in shaping AMF community structures at regional scale, we examined AMF communities associated with dominant plant species along a precipitation gradient in Tibetan alpine steppe. Rhizosphere soils were collected from five sites with annual precipitation decreasing from 400 to 50 mm. A total of 31 AMF operational taxonomic units (OTUs) were identified. AMF community composition varied significantly among sites, whereas AMF community composition did not vary among plant species. Path analysis revealed that precipitation directly affected AMF hyphal length density, and indirectly influenced AMF species richness likely through the mediation of plant coverage. Our results suggested that water availability could drive the changes of AMF communities at regional scale. Given the important roles AMF could play in the dynamics of plant communities, exploring the changes of AMF communities along key environmental gradients would help us better predict the ecosystem level responses of the Tibetan vegetation to future climate change.

The Tibetan Plateau, as the “the third pole of the world”, is the highest plateau in the world with an average altitude of 4 000 m above sea level (a.s.l)^1^. Because of its unique geographic settings and harsh climatic conditions characterized by high ultraviolet radiation, strong wind, poor nutrient soil and low temperature^2^, the Tibetan Plateau is recognized as one of the most challenging habitats for both aboveground and belowground organisms. As a consequence, the alpine grassland ecosystems that dominate this region are relatively low in species diversity and more susceptible to climate change compared to grasslands in other regions. In fact, anthropogenic climate change is predicted to have severe impacts on precipitation patterns and lead to increased drought stress on the Tibetan Plateau^3^,^4^. Significant progress has been made in the past decade to address adaptation mechanisms of aboveground vegetation to global change^3^,^5^,^6^. However, we still know little about how belowground communities would respond to changes of key climatic variables^7^,^8^, which limits our ability to predict the ecosystem level responses of the Tibetan Plateau to future climate change^9^,^10^.

As one of the most widespread and ecologically important groups of belowground organisms, arbuscular mycorrhizal fungi (AMF, Phylum Glomeromycota) form mutualistic associations with the majority of alpine grassland plants on the Tibetan Plateau^11^,^12^,^13^. The plants provide photosynthetic carbon for the growth and functioning of AMF. In return, AMF help host plants with better access to soil nutrients and increased resistance to stress and pathogens^14^,^15^,^16^. Because AMF have important roles in influencing plant growth and fitness, driving community dynamics and maintaining ecosystem stability^17^,^18^,^19^, it is essential to examine their community changes in response to altered climatic conditions.

The climate of the Tibetan Plateau is mainly dominated by a monsoon wind system which causes annual precipitation to vary greatly in the region especially where the alpine steppe occupies^1^,^20^. Changes in water availability, both temporarily and spatially, are likely to have both direct and indirect effects on the community composition of AMF. Soil moisture may influence AMF community assembly within roots by regulating the level of AMF colonization and the assemblage of different species^21^,^22^. Variability in water regimes can also directly affect the growth and distribution of extraradical mycelium in the soil^23^,^24^,^25^. Alternatively, altered precipitation patterns can indirectly affect AMF via the changes in the above-ground plant community composition, coverage, diversity and productivity^26^,^27^,^28^. The ability of different plant-AMF combinations to cope with drought stress varies. When water is limited, plants may favor AMF species that produce more extra-intraradical hyphae in order to increase water absorption. It was documented that certain AMF species lost the ability to colonize plants under drought conditions^21^. When soil water is abundant, AMF species that are more efficient for supplying water but more demanding for carbon might be abandoned by host plants according to the functional equilibrium models^29^,^30^. A number of studies have reported the effects of drought on AMF abundance and diversity^31^,^32^,^33^, but to our knowledge there is no report on whether or how precipitation could affect AMF communities on the Tibetan Plateau at regional scales.

The alpine grasslands mainly include alpine steppe and alpine meadow, which cover more than 60% of the land on the Tibetan Plateau. Alpine steppe alone occupies about 29.1% of the grassland area in this region^34^. *Stipa purpurea* is an endemic constructive species of alpine steppe and widely distributed in the arid and semi-arid areas above 4000 m a.s.l. This species is a perennial mycorrhizal grass and can grow well in alpine adverse environments because of its high resistance to cold, drought and strong winds. It also serves as forage for grassland animals and plays an important role in safeguarding water and stabilizing soils. Meanwhile, the frequently accompanying species *Potentilla bifurca* L. and *Leontopodium nanum* were also mycorrhizal plants. In the past several years, a relatively high AMF diversity has been documented in the Tibetan grasslands^11^,^13^,^35^,^36^,^37^. However, these studies were carried out in scattered and discontinuous areas, or by controlled experiments.

In this study, we chose to study AMF associated with three dominant plant species, *S. purpurea*, *P. bifurca* and *L. nanum*, at five sites spanning across the entire region of the Tibetan alpine steppe with a precipitation gradient ranging from 400 mm to 50 mm. We used molecular techniques to characterize AMF communities to address the following main questions: (1) whether AMF communities would change along this precipitation gradient? (2) If yes, would the variation in precipitation contribute to the observed changes in AMF communities?

## Results

### Community composition of AMF

**Figure 1:**
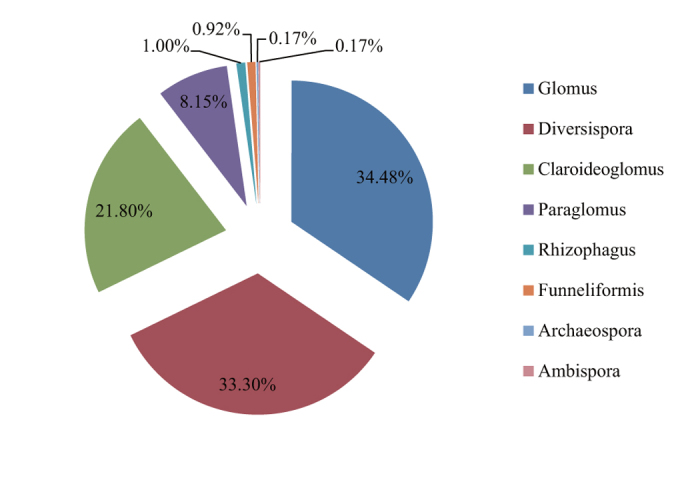
Relative abundance (%, percentage of clones) of AMF genera detected in rhizosphere soil of Tibetan alpine steppe.

A total of 31 different operational taxonomic units (OTUs) were identified from rhizosphere soils based on 2294 sequenced AMF clones (Fig. S1). They belonged to eight genera including *Glomus*, *Diversispora*, *Claroideoglomus*, *Paraglomus*, *Rhizophagus*, *Funneliformis*, *Archaeospora* and *Ambispora*. The most dominant genera were *Glomus, Diversispora* and *Claroideoglomus*, representing approximately 34.5%, 33.3% and 21.8% of the total clones, respectively (Fig. 1). Out of the 31 AMF OTUs detected in the present study, 25 matched well with molecular virtual taxa that had been detected in other regions and recorded in the Maarj*AM* database (Table S1). The other six OTUs that were infrequently found in the field did not match any recorded molecular virtual taxa in this database.

The detected OTUs were not distributed evenly among host plants and collection sites. The numbers of OTUs associated with plant species *S. purpurea*, *L. nanum* and *P. bifurca* were 26, 19 and 17, respectively (Table S1). Nine OTUs were shared by all three plant species. OTUs *Div*-4 (22.7% of all AMF clones) and *Glo*-6 (19.3%) were most frequently found in all plant species and in all sampling sites. Some of the less frequent OTUs were only detected in soils either from in one or two sites (such as *Amb*-1, *Glo*-12 and *Arc*-1) or from a portion of host-site combinations (such as *Par*-2, *Clar*-1 and *Clar*-4).

### AMF colonization, diversity and composition along the precipitation gradient

Root colonization percentages (%RLC) of *S. purpurea* in NM and GG were significantly higher than that in other three sampling sites (Table 1). AMF hyphal length density (HLD) varied significantly among sites (*F* = 13.43, *P* < 0.001) and the highest HLD occurred in the rhizosphere of *S. purpurea* and *L. nanum* in NM. AMF species richness per sample was relatively low in our study (6 ± 0.24), and varied significantly among sampling sites (*F* = 9.52, *P* < 0.001) but not among plant species (*F* = 2.18, *P* = 0.12). The two-way analysis of variance (ANOVA) showed that sampling site had significant effects on AMF Shannon–Wiener index (*F* = 2.73, *P* = 0.039, Table 1). One-way ANOVA revealed that AMF phylogenetic diversity in the rhizosphere of *S. purpurea* and Shannon–Wiener index in *S. purpurea* and *L. nanum* were significant different among the sites (Table 1).

**Figure 2:**
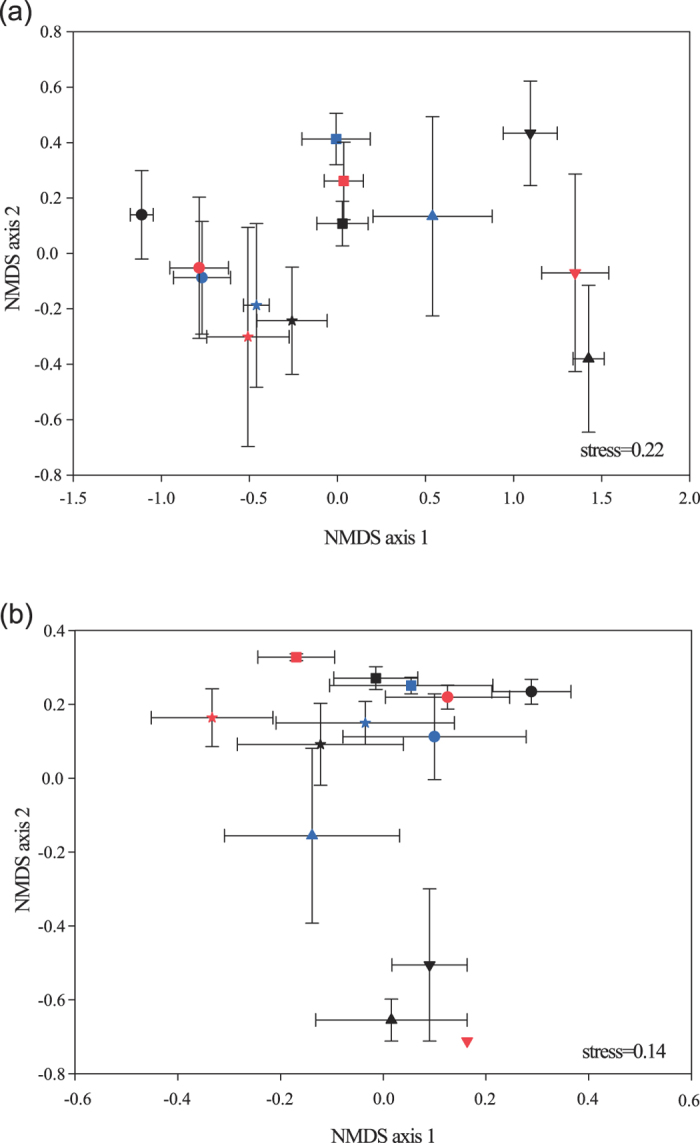
Nonmetric multidimensional scaling (NMDS) plots demonstrating the effects of sampling sites on AMF communities along the precipitation gradient in the Tibetan alpine steppe using the data of (**a**) taxonomic composition (Bray–Curtis) and (**b**) phylogenetic composition (Unifrac distance). Each point represents the centroid of the AMF community of each plant per species with vertical and horizontal bars depicting ±SE. The color of black, red and blue refers to *S. purpurea*, *L. nanum* and *P. bifurca*, respectively. •, ★, ▄, ▴ and ▾ represent the sampling sites BG, NM, ZC, GZ and GG, respectively.

As shown on the non-metric multidimensional scaling (NMDS) plots, AMF communities from different sites were grouped separately (Fig. 2a, based on the relative abundance of OTUs per sample; Fig. 2b, based on phylogenetic compositions). This pattern was verified by the MRPP results (Table S2) and AMOVA (*F* = 2.47, *P* = 0.005). However, AMF communities associated with different plant species from the same site hosted similar compositions and there were no significant differences between AMF communities of any pair of host plants within a site (Table S3).

### Relationships among AMF, precipitation, soil and plant properties

Soil organic matter (SOM), total nitrogen (N), pH, and the contents of clay, silt and sand were all significantly different among sampling sites (Table S4). Soil phosphorus (P) content, ranging from 4.32 to 8.8 mg/kg, varied significantly among host plant species (*F* = 3.59, *P* = 0.03), but not among sampling sites (*F* = 1.76, *P* = 0.14). Plant coverage (*r* = 0.82, *P* < 0.001), soil total N (*r* = 0.41, *P* = 0.001), SOM (*r* = 0.59, *P* < 0.001), silt content (*r* = 0.41, *P* = 0.001) and clay content (*r* = 0.38, *P* = 0.003) were positively correlated with precipitation.

**Figure 3:**
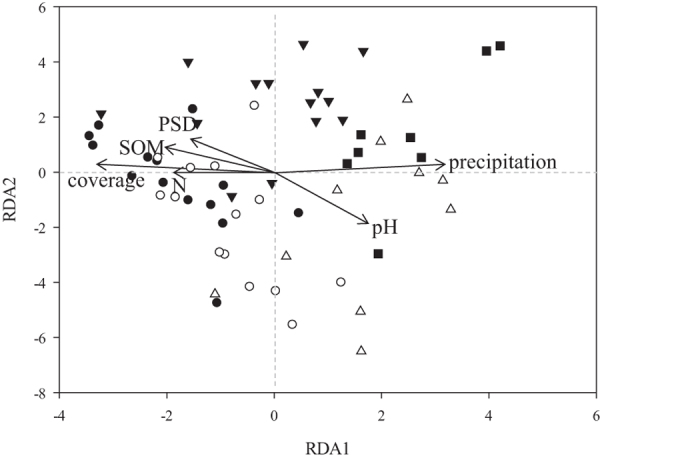
Redundancy Analysis (RDA) plot of AMF community composition in rhizosphere of three plant species and vectors of significant (*P *< 0.05) environmental variables across sites. The first and second axes explain 20.69% and 4.19% of the variance, respectively. •, Ł, ▾, ▵ and ▄ represent the sampling sites BG, NM, ZC, GZ and GG, respectively.

**Figure 4:**
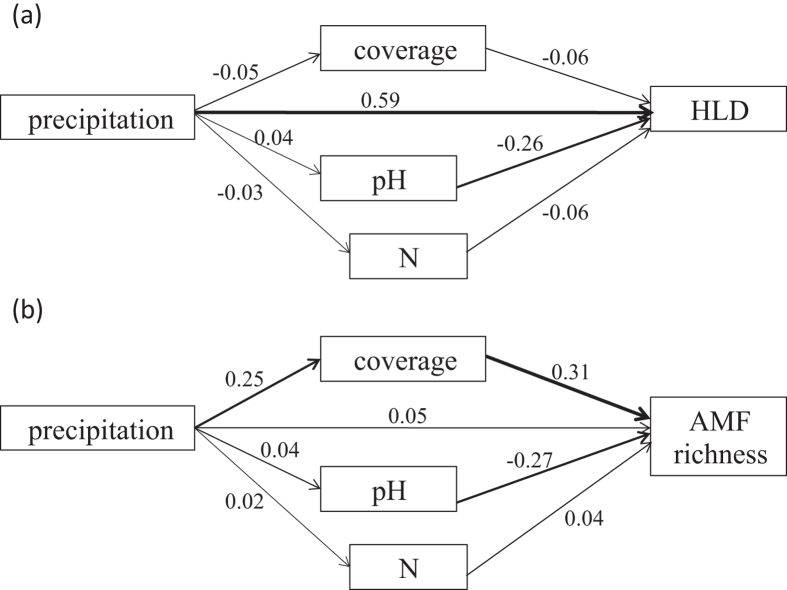
Path diagrams showing the direction and magnitude of the influences of precipitation on (**a**) AMF hyphal length density (HLD) and (**b**) AMF species richness.

Precipitation, soil variables (SOM, pH, total N and PSD) and plant coverage were found to have significant effects on AMF communities based on the results of Mantel test and partial Mantel test (Table S5), and RDA on relative abundance of OTUs showed that these variables explained a total of 30% of the variance in the composition of AMF communities among sites (*F* = 3.84, *P* = 0.001, Fig. 3). When controlling other environmental factors, precipitation showed the strongest effect on AMF community composition (*r* = 0.46, *P* = 0.001), while plant coverage, pH and total N showed less but significant influences (Table S5). AMF species richness and HLD were positively correlated with precipitation, SOM and plant coverage, and negatively correlated with soil pH. Moreover, AMF species richness showed positive relationship with soil total N content significantly (Table S6). We used path analysis to further examine the relative contributions of these variables to AMF species richness and HLD. As shown in Fig. 4, precipitation (path coefficient = 0.59) was the most significant factor that directly affected HLD. Precipitation showed weak direct effect on AMF species richness, but it significantly changed plant coverage (path coefficient = 0.25) which had substantial influence on AMF species richness (path coefficient = 0.31). Soil pH also had significant influences on AMF species richness (path coefficient = −0.27) and HLD (path coefficient = −0.26).

## Discussion

To our best knowledge, the study presented here is the first regional scale investigation focusing on the changes of AMF communities along a natural precipitation gradient in the Tibetan alpine steppe. Our results showed that AMF community composition varied significantly among sites with different amounts of precipitation, suggesting that water availability might be a possible driver of AMF assemblages in this region. Although previous studies have reported that host identity strongly affected the composition of AMF communities^38^,^39^, we did not observe significant host specificity of AMF in any of five sites based on either the relative abundance of OTUs per sample or phylogenetic composition. In addition, HLD and AMF species richness showed a decline with the decreasing precipitation. Previous studies have reported that drought decreased AMF abundance^22^,^27^ and the increased precipitation enhanced AMF abundance in three types of grassland in Tibetan Plateau^35^. However, contrasting results were also obtained which showed that AMF community or root colonization was not affected by drought^40^. The changes of community composition may be due to different abilities among AMF species to adapt to drought conditions^10^.

Besides precipitation, plant coverage, soil pH and total N could also be important driving factors for shaping AMF communities in the Tibetan alpine steppe. Lower AMF diversity observed in sites with less coverage of vegetation is likely because the reduced amount of carbon allocated to belowground could only support a less diverse community of fungi when the total carbohydrates photosynthesized by plants become less^41^,^42^. Soil pH may exert a direct effect on AMF community composition or an indirect effect because of its impact on P availability^43^. Since soil P content did not vary among the sites, it is more likely that pH affected AMF communities directly than indirectly through the changes on P availability. As the most prominent factor that determines the occurrences and functioning of AMF, soil P showed no relationship with AMF community variation in our sites, partially because soil P contents in this region do not seem to be a limiting factor for plant growth according to previous reports^44^,^45^. However, we found soil total N contents varied significantly among sites and were significantly correlated with the variation in AMF community composition. Whether soil N content has the direct influence on the community composition of AMF in this region remains unknown, which raises interesting opportunities for further work.

Significant relationships between AMF species richness, HLD and environmental factors, such as precipitation, plant coverage, SOM and pH, were revealed by correlation analysis. Moreover, AMF species richness was also correlated with N, silt and sand contents. However, among these factors, precipitation contributes most to the differences in some of the key mycorrhizal variables such as HLD while plant coverage was the strongest predictor for the changes of AMF richness. The extensive AMF hyphal network in the soil provides an important pathway for the flow of nutrients and water from soil into the roots^15^ and water uptake may be more important under drier soils. On one hand, soil moisture may determine AMF hyphal turnover rates which range from a few hours to several days, and possibly even longer during drought^46^ and thus determines the standing crop of hyphae in the soil^31^. On the other hand, drought has been reported to reduce the allocation of photosynthates to plant rhizosphere and to negatively impact the formation of myceli^10^. Our results showed that plant coverage was positively correlated with AMF species richness, indicating that water availability may determine aboveground productivity and further affect AMF abundance and diversity. Moreover, the positive correlations between SOM and plant biomass and coverage suggest that host plants might allocate more photosynthates to AMF which play more important role in stimulating soil carbon pools to support plant growth^16^,^19^.

In recent years, increasing attention has been paid to the investigations of AMF diversity in the Tibetan alpine ecosystem^38^,^47^,^48^. A total of 31 AM fungi phylotypes detected in this study are much higher than the number of 23 morphospecies detected in the southern Tibetan Plateau^35^ and 21 phylotypes (from roots and spores) in five study sites of the central Tibet Plateau^38^. It is well documented that AMF species vary in their adaptability to different environments^21^,^22^,^26^ and the species with better adaptability to an environment is expected to replace those that are poorly adapted. We found *Glomus* species were gradually replaced by *Diversispora* with decreasing precipitation (Fig. S2), suggesting that *Glomus* may be poorly adapted to drought. Yang *et al.*^49^ also found *Glomus* species were replaced by other species under dry conditions by examining the community structures of AMF in Grasslands National Park, Canada. Changes in the composition of AMF communities can potentially feedback to influence the dynamics of aboveground communities and ecosystem stability^50^,^51^. Further work is necessary to include the variation in AMF communities along key environmental gradients in order to better predict the ecosystem level responses of the Tibetan vegetation to future climate change.

## Materials and Methods

### Study area and sampling

**Figure 5:**
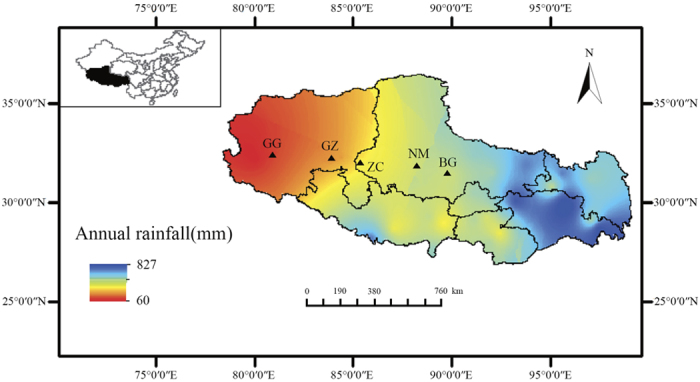
Geographic locations of the five sampling sites and the regional patterns of precipitation. Abbreviations of site names (same as described in Table 2) are shown on the map. Map was created with ArcGIS 10.0 (http://www.esri.com/software/arcgis/, ESRI, Redlands, CA, USA).

The typical alpine steppe in the northern Tibetan Plateau is mainly located between 31 and 33°N latitude and 80° and 90°E longitude where the average altitude is above 4400 m a.s.l. It is situated in the high-cold semi-arid and arid zone and covers a total area of 60 × 10^4 ^km^2^, with an average annual temperature between −3 and 6 °C and an average annual precipitation ranging from 50 to 400 mm. Approximately 80–90% of the precipitation occurs during the months of June to August, while the growing season extends from mid-May to late September. The soil is classified as alpine steppe soil (Sandy loamy) with relatively low soil organic carbon and soil mineral nutrients. The plant community is dominated by *S. purpurea* with coverage about 20–40%. *L. nanum* and *P. bifurca* are two of the most common accompanying species. According to the annual precipitation data from 39 climatic stations (China Meteorological Data Sharing Service System; http://cdc.cma.gov.cn/) across the Tibetan Plateau during the period of 2003–2012 using the Kriging interpolation, the precipitation decreases from 400 mm in southeast of this region to below 50 mm in northwest. In this study, we chose five sites that are distributed evenly across the precipitation gradient on the typical alpine steppe (Fig. 5). The location and characteristics of the five sampling sites are listed in Table 2.

Sample collection was carried out in early August 2014 when plant biomass peaks in this region. Three perennial plant species on the alpine steppe, *S. purpurea*, *L. nanum* and *P. bifurca* were targeted in each site except GG and GZ. There were few *P. bifurca* in GG and few *L. nanum* in GZ, so we only sampled two other species in these two sites. At each sampling site, five 1 × 1 m^2^ quadrats within an area of 100 m^2^ were randomly selected. Prior to the sampling of rhizosphere soil, plant species coverage of different species was recorded. In each quadrat, three closest individuals of *S. purpurea*, *L. nanum* or *P. bifurca* were chosen. Rhizosphere soil under each individual plant was carefully excavated to approximately 20 cm depth from ground surface, and soils collected from the same species were mixed as one sample. Roots and soil were separated stored in plastic bags at 4 °C in a refrigerated box before being transported to laboratory. The shoots of each chosen plant were also collected during field sampling and oven-dried (65 °C for 48 h) for the measurement of plant biomass. A subset of roots from each sample were washed and stored at −20 °C for the assessment of AMF colonization. Approximately 100 g fresh soil was air-dried and passed through a 2 mm sieve for analyzing soil physical and chemical properties and extracting extraradical hyphae. Another 50 g fresh soil was kept at −80 °C for molecular analysis of AMF communities.

### Soil physical and chemical properties

Soil pH was determined in a 10 g subsample of air-dried and 2 mm sieved soil with a soil: water ratio of 1:2.5 (w/v). Soil available P was extracted with 0.5 M NaHCO_3_ and measured by anti-spectrophotometry method. SOM concentration was measured by oxidization with K_2_Cr_2_O_7_. Total N content in the soil was analyzed using an elemental analyzer. Soil particle size distribution (PSD) was analyzed using a laser diffraction technique with a Longbench Mastersizer 2000. Soil particle sizes from 0 to 2000 μm were divided into seven classes and soil PSD was characterized by the volume fractal dimension value (D value)^52^.

### AMF colonization and extraradical hyphae

Due to the limited quantity of roots of the other species, we were only able to examine mycorrhizal colonization of *S. purpurea* in this study. Roots were washed carefully with tap water and cut into segments of *c*. 1 cm length. Approximately 100 root segments were randomly chose and cleared in 10% KOH at 90 °C and stained with 0.05% Trypan blue. Roots segments were randomly selected and examined for the percentage of root length colonized by AMF (%RLC) using the magnified intersection method at 200× magnification^53^. The extraradical hyphae were extracted from soil by the membrane filter technique^54^ and stained with Trypan blue using the methods of Brundrett *et al.*^55^. HLD (m/g dry soil) was measured by the grid intercept method at 200× magnification.

### DNA extraction and PCR

DNA was extracted from 0.5 g of soil samples (fresh weight) using the PowerSoil^®^ DNA Isolation Kit (MoBio Laboratories, Inc., Carlsbad, CA, USA) according to the instructions of the manufacturer. The quality and quantity of the extracted DNA were determined by electrophoresis on a 1.0% agarose gel and spectroscopic analysis (NanoDrop Technologies, Wilmington, DE, USA). DNA extracts were stored at −20 °C and then 10-fold diluted DNA as templates of subsequent PCRs.

Partial small subunit (SSU) ribosomal RNA gene fragments were amplified using nested PCR. DNA was first amplified using the general eukaryotic primers GeoA2 and Geo11 to amplify a *c*. 1.8 kb fragment of the 18S rRNA gene^56^. PCR was carried out in a final volume of 25 μl consisting of 2 μl extracted DNA dilution and 1 μl (10 μM) of each primer using the 2× Taq PCR mastermix system (Tiangen Biotech) with the following cycling conditions: initial denaturation at 94 °C for 3 min, followed by 30 cycles at 94 °C for 30 s, 59 °C for 1 min, 72 °C for 2 min, and a final extension period at 72 °C for 10 min.

An aliquot of 2 μl of the first PCR product was diluted 1/100 with ddH_2_O and used as template for the second PCR reaction using primers NS31 and AML2^57^,^58^. The second PCR was carried out in a final volume of 50 μl with the following cycles: 94 °C for 3 min, followed by 30 cycles at 94 °C for 30 s, 58 °C for 1 min, 72 °C for 1 min, and a final extension period at 72 °C for 10 min. Second-step PCR products were visualized on a 1.0% agarose gel containing GelRed^TM^ (Biotium) to confirm the success of amplification.

### Cloning and sequencing

The PCR products were purified using PCR Product Gel Purification Kit (Axygen, Union City, CA, USA). Purified DNA was cloned into the pGEM-T Easy Vector System (Promega, USA) and then transformed into competent cells of *Escherichia colistrain* DH-5α (Tiangen Biotech) following the manufacturer’s protocols. A total of 61 clone libraries were obtained. For each library, 50 positive colonies were picked and grown overnight in liquid Luria-Bertani (LB) medium. All clones were sequenced using the vector primer T7 on an ABI 3730 DNA analyzer. Representative sequences from each encountered AM fungal OTU in this study have been deposited in the GenBank with accession numbers KT238940-KT238970.

### Sequence analysis

All the DNA sequences were edited and compared with the GenBank database through BLASTN on the NCBI website (http://blast.ncbi.nlm.nih.gov/). Non-Glomeromycota and unqualified sequences were eliminated from the dataset. All sequences that were unambiguously identified as AMF were first aligned using ClustalW, and then grouped into different OTUs using a similarity of 97% with the program Mothur^59^. We selected one sequence to represent each OTU and aligned representative sequences together with the reference sequences of all major Glomeromycota clades using ClustalW algorithm. Neighbor-joining (Kimura 2-parameter model with 1000 bootstrap replications) phylogenetic analyses were computed in software MEGA 6.06^60^. The tree was rooted with *Mortierella polycephala* (accession no. X89436) and *Endogone pisiformis* (accession no. X58724)^58^.

We used the online Maarj*AM* database (http://maarjam.botany.ut.ee; Öpik *et al.*, 2010; status on 12 April 2015) to group the representative sequence per OTU obtained in this study into corresponding molecular virtual taxa with the sequence identity ≥98%^38^ in order to compare our OTUs with the AMF detected in other ecosystems.

### Statistical analysis

The relative abundance of each OTU in a community was calculated as the percentage of clone sequences grouped into that OTU within a sample. AMF species richness was counted as the numbers of OTUs in a sample. To compare α-diversity between samples, we calculated Shannon-Wiener index and phylogenetic diversity (PD) for each sample in Mothur. One-way ANOVA were performed to examine the differences in each variable among plant species or sampling sites respectively, and the significant differences were determined with Duncan’s multiple comparison tests at the 95% confidence level. General Linear Model (GLM) was performed to test the main effects of sampling site and plant species on observed parameters using the SPSS 17.0 software package. All AMF community-related analyses were based on the relative abundance of OTUs per sample. Data on AMF community composition were plotted using a Nonmetric Multidimensional Scaling (NMDS) ordination with Bray-Curtis distance measurements and the function ‘monoMDS’ from the vegan package in R (version 3.1.2). The differences in the AMF communities among different sites or host species within each site were tested using the analysis of Multiple Response Permutation Procedure (MRPP). The MRPP delta values measure the overall weighted indicate of group mean distances and *P* values mean the significance of the test. In addition, we analyzed the phylogenetic composition structure with weighted Unifrac distance using the Mothur to test whether there were phylogenetically clustered patterns within sites or host species and then an analysis of molecular variance (AMOVA) was performed to test the significant differences in phylogenetic composition among sites or host species. The relationships between environmental factors and AMF communities were assessed using Mantel test and redundancy analysis (RDA) in R. To control the covarying effects of environmental factors, partial Mantel test was also carried out with each of the significant independent variables according to the result of Mantel test.

Path analysis is an extension of multiple linear regressions to provide estimates of the magnitude and significance of hypothesized causal connections among variables^61^. We performed path analysis to estimate the direct and indirect effects of precipitation on AMF species richness and HLD using SPSS 17.0. Prior to the procedure, a correlation analysis among the variables was conducted and only variables that were significantly correlated to AMF species richness and HLD were included in the model. All environmental factors were log transformed to achieve normality.

## Additional Information

**How to cite this article**: Zhang, J. *et al.* Precipitation shapes communities of arbuscular mycorrhizal fungi in Tibetan alpine steppe. *Sci. Rep.* **6**, 23488; doi: 10.1038/srep23488 (2016).

## Supplementary Material

Supplementary Information

## Figures and Tables

**Table 1:** The percentage of root length colonized by AMF (%RLC), AMF hyphal length density (HLD), AMF specie richness, AMF phylogenetic diversity (PD) and Shannon–Wiener index (mean ± standard error) in rhizosphere soil of *S. purpurea*, *L. nanum* and *P. bifurca* along the precipitation gradient in Tibetan alpine steppe.

Plant/site	RLC(%)	HLD (m/g)	AMF specie richness	PD	Shannon–Wiener index
*S. purpurea*					
BG	7 ± 3a	15.33 ± 4.03bc	6.46 ± 0.47b	0.44 ± 0.04b	1.02 ± 0.20a
NM	34 ± 8b	17.92 ± 1.48bc	7.6 ± 0.85b	0.51 ± 0.05b	1.64 ± 0.07c
ZC	10 ± 2a	10.44 ± 1.56ba	6.84 ± 0.47b	0.46 ± 0.02b	1.60 ± 0.10bc
GZ	5 ± 4a	5.63 ± 1.44a	3.8 ± 0.85a	0.28 ± 0.02a	1.11 ± 0.05ab
GG	31 ± 8b	8.05 ± 1.55a	7.22 ± 0.71b	0.48 ± 0.09b	1.23 ± 0.26abc
*L. nanum*					
BG	/	14.23 ± 2.46b	6.65 ± 0.55b	0.59 ± 0.12a	1.52 ± 0.12b
NM	/	17.64 ± 2.62b	7.13 ± 0.91b	0.39 ± 0.05a	1.33 ± 0.11b
ZC	/	4.21 ± 0.86a	6.84 ± 0.47b	0.44 ± 0.03a	1.51 ± 0.06b
GG	/	2.42 ± 0.24a	3.17 ± 0.63a	0.34 ± 0.12a	1.04 ± 0.08a
*P. bifurca*					
BG	/	14.73 ± 2.68b	7.6 ± 0.6b	0.45 ± 0.03a	1.37 ± 0.14a
NM	/	9.07 ± 1.83ab	7.6 ± 0.00b	0.51 ± 0.02a	1.56 ± 0.08a
ZC	/	8.62 ± 1.75b	6.84 ± 0.47b	0.46 ± 0.03a	1.33 ± 0.12a
GZ	/	5.39 ± 1.28a	4.56 ± 0.76a	0.48 ± 0.08a	1.33 ± 0.17a
site	/	*	*	–	*
plant species	/	–	–	–	–

Data with different lowercase letters and “*” Indicate significant levels at *P* < 0.05. “–” Indicates no significance. “/” Means no data available.

**Table 2:** Location and characteristics of the five sampling sites along the precipitation gradient.

Sampling sites	Latitude	Longitude	Altitude (m)	Annual precipitation (mm)	Annual temperature (°C)	Vegetation type
Baingoin (BG)	31°31′N	89°46′E	4499	365.13	2.33	Alpine steppe
Nyima (NM)	31°52′N	88°15′E	4621	382.49	1.61	Alpine steppe
Zhongcang (ZC)	32°01′N	85°23′E	4824	200.66	2.27	Alpine steppe
Gêrzê (GZ)	32°18′N	83°58′E	4413	171.00	2.33	Alpine steppe
Gê'gya (GG)	32°27′N	80°56′E	4490	59.96	0.87	Alpine desert

Data of average annual precipitation and mean annual temperature are based on the years of 2003–2012. Abbreviations of site names are shown in parentheses.
