# Corrigendum: Precipitation shapes communities of arbuscular mycorrhizal fungi in Tibetan alpine steppe

**DOI:** 10.1038/srep24556

**Published:** 2016-04-22

**Authors:** Jing Zhang, Fang Wang, Rongxiao Che, Ping Wang, Hanke Liu, Baoming Ji, Xiaoyong Cui

Scientific Reports 6: Article number: 23488; 10.1038/srep23488published online: 03222016; updated: 04222016

In this Article, Affiliation 2 was omitted for Jing Zhang. The correct affiliation is listed below:

College of Forestry, Beijing Forestry University, No. 35, Tsinghua East Road, Haidian District, Beijing, 100083, China.

