# Supplementary material for: Precipitation shapes communities of arbuscular mycorrhizal fungi in Tibetan alpine steppe
Source: Sci Rep. 2016 Mar 22;6:23488. doi: 10.1038/srep23488 (PMC4802204; doi:10.1038/srep23488)
Supplement: Supplementary Information [file srep23488-s1.pdf]

Supplementary Information

Precipitation shapes communities of arbuscular mycorrhizal fungi in Tibetan alpine steppe

Jing Zhang [1], Fang Wang [1], Rongxiao Che [1], Ping Wang [2], Hanke Liu [1], Baoming Ji [2*]
Xiaoyong Cui [1*]

[1] College of Life Sciences, University of Chinese Academy of Sciences, No. 19A, Yuquan Road, Shijingshan District, Beijing, 100049, China

[2] College of Forestry, Beijing Forestry University, No.35, Tsinghua East Road, Haidian District, Beijing, 100083, China

*Corresponding authors: Baoming Ji, Xiaoyong Cui

Tel: +86-10-88256497; Fax: +86-10-88256497

E-mail: baomingji@bjfu.edu.cn, cuixy@ucas.ac.cn

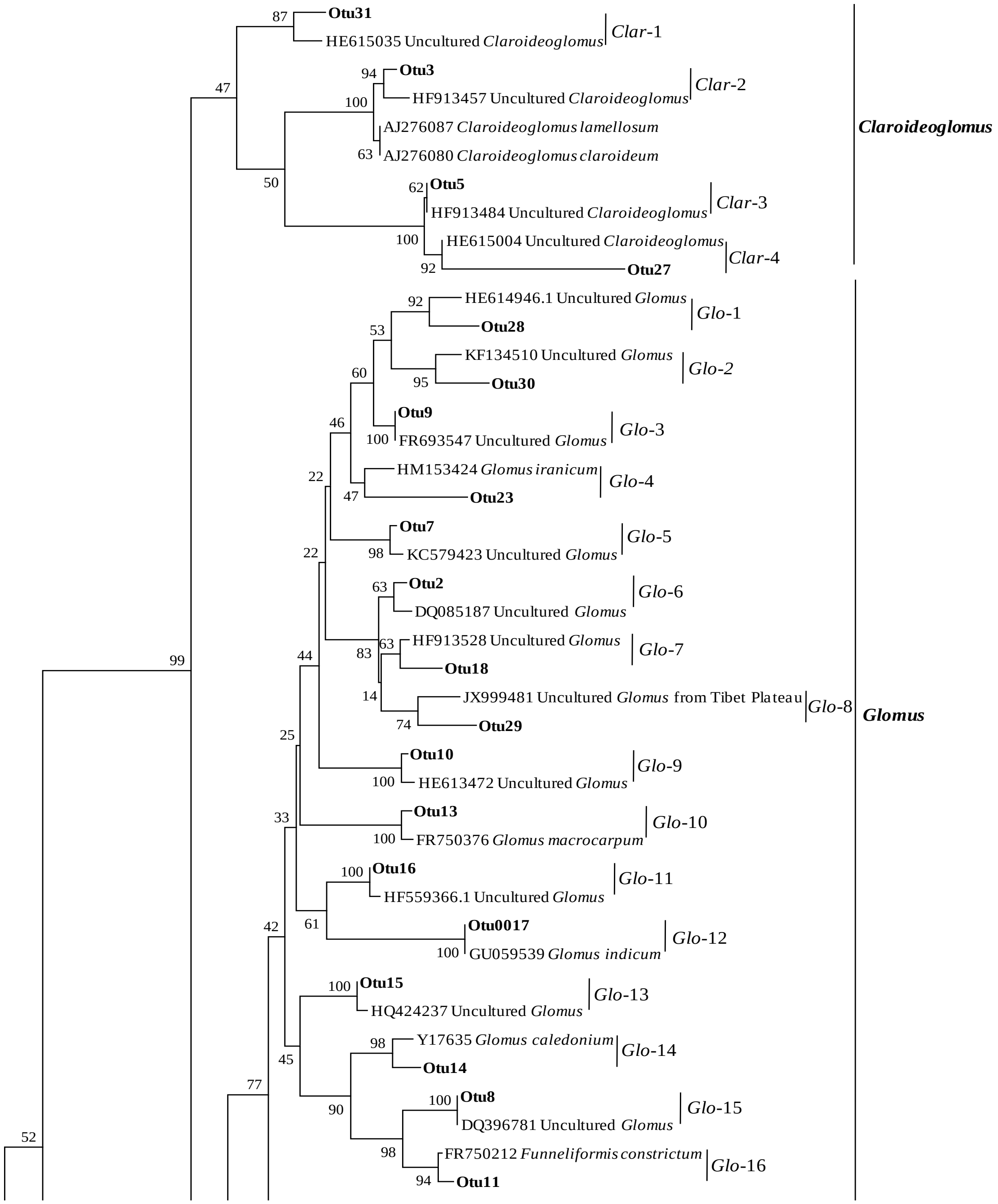

Otu31
HE615035 Uncultured Claroideoglomus
Clar-1
Otu3
HF913457 Uncultured Claroideoglomus
Clar-2
AJ276087 Claroideoglomus lamellosum
AJ276080 Claroideoglomus claroideum
Claroideoglomus
Otu5
HF913484 Uncultured Claroideoglomus
Clar-3
HE615004 Uncultured Claroideoglomus
Otu27
Clar-4
HE614946.1 Uncultured Glomus
Otu28
Glo-1
KF134510 Uncultured Glomus
Otu30
Glo-2
Otu9
FR693547 Uncultured Glomus
Glo-3
HM153424 Glomus iranicum
Otu23
Glo-4
Otu7
KC579423 Uncultured Glomus
Glo-5
Otu2
DQ085187 Uncultured Glomus
Glo-6
HF913528 Uncultured Glomus
Otu18
Glo-7
JX999481 Uncultured Glomus from Tibet Plateau
Otu29
Glo-8
Glomus
Otu10
HE613472 Uncultured Glomus
Glo-9
Otu13
FR750376 Glomus macrocarpum
Glo-10
Otu16
HF559366.1 Uncultured Glomus
Glo-11
Otu0017
GU059539 Glomus indicum
Glo-12
Otu15
HQ424237 Uncultured Glomus
Glo-13
Y17635 Glomus caledonium
Otu14
Glo-14
Otu8
DQ396781 Uncultured Glomus
Glo-15
FR750212 Funneliformis constrictum
Otu11
Glo-16
87 94 100 63 47 50 62 100 92 99 92 53 95 60 100 46 47 22 22 98 63 83 63 14 74 44 100 25 100 33 100 61 100 42 100 98 45 100 77 90 98 94 52

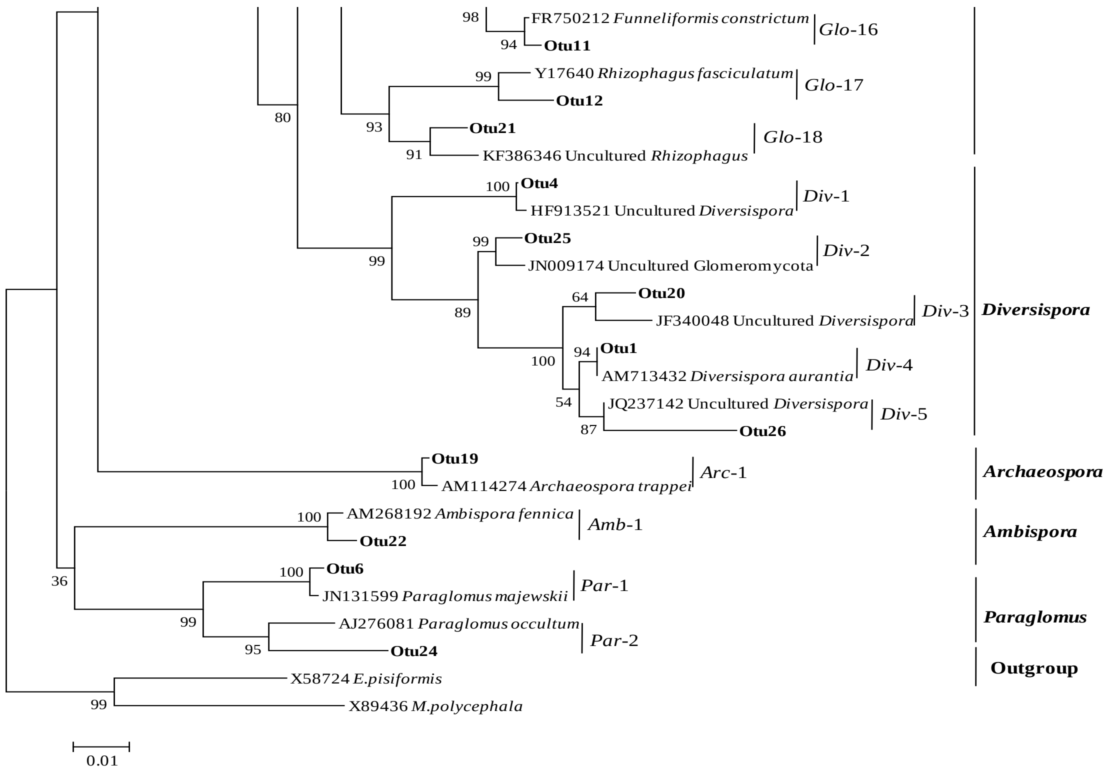

Figure S1. Neighbor joining phylogenetic tree of the Glomeromycota based on partial SSU rDNA sequences showing the phylogenetic relationship of the OTUs obtained from this study. Numbers above the branches denote bootstrap values from 1000 replications (values＞60% are shown).

Figure S2. Relative abundance (%, percentage of clones) of AMF genera of rhizosphere soil in each site.

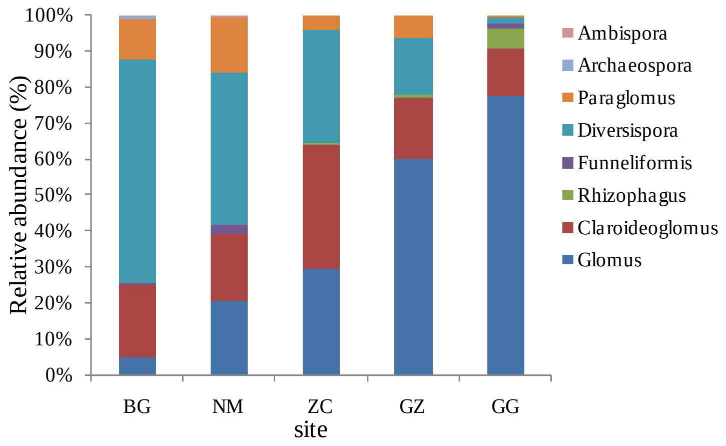

Table S1. Number of clones for each AMF OTU detected in all combinations of plant species and sampling sites and each OUT's most closely related molecular virtual taxon recorded in the Maarj*AM* database (http://maarjam.botany.ut.ee/)

OTU	*S. purpurea*					*L. nanum*				*P. bifurca*				Molecular virtual taxon	
	BG	NM	ZC	GZ	GG	BG	NM	ZC	GG	BG	NM	ZC	GZ	Maarj*AM*	Identity
Glo-1	0	0	0	0	0	0	0	0	0	0	1	0	0	VTX00342	98%
Glo-2	0	0	0	0	1	0	0	0	0	0	0	0	0	VTX00166	97%
Glo-3	0	3	6	0	10	0	0	0	4	2	2	3	4	VTX00342	99%
Glo-4	0	0	1	0	0	0	0	0	0	0	0	0	1	VTX00155	96%
Glo-5	0	21	0	32	15	0	0	0	29	25	9	0	0	VTX00304	99%
Glo-6	1	21	57	45	105	11	15	43	28	4	11	53	49	VTX00143	99%
Glo-7	0	0	2	1	2	0	0	0	1	0	0	0	0	VTX00143	98%
Glo-8	0	0	0	0	1	0	0	1	0	0	0	0	0	VTX00143	97%
Glo-9	0	0	0	0	7	0	0	0	16	0	0	0	0	VTX00174	99%
Glo-10	0	0	0	10	2	0	0	0	0	1	0	0	2	VTX00199	99%
Glo-11	0	0	0	0	0	3	0	0	0	1	0	0	7	VTX00130	99%
Glo-12	0	0	0	6	0	0	0	0	0	0	0	0	1	VTX00222	100%
Glo-13	0	4	0	4	1	0	0	0	4	0	0	0	0	VTX00301	99%
Glo-14	0	0	0	13	0	0	0	0	0	0	0	0	0	VTX00067	100%
Glo-15	0	1	0	36	0	0	0	0	0	0	26	0	24	VTX00293	100%
Glo-16	0	0	0	0	4	0	15	0	0	2	0	0	0	VTX00064	99%
Glo-17	0	0	0	0	16	0	0	3	0	0	0	0	0	VTX00105	99%
Glo-18	0	0	0	4	0	0	0	0	0	0	0	0	0	VTX00214	98%
Clar-1	0	0	0	0	1	0	0	0	0	0	0	0	0	VTX00193	98%
Clar-2	2	9	16	29	27	4	3	37	11	12	20	47	45	VTX00193	99%
Clar-3	16	11	28	0	0	29	46	55	0	27	10	14	0	VTX00056	99%
Clar-4	0	0	0	0	0	0	1	0	0	0	0	0	0	VTX00056	96%
Div-1	32	42	3	1	0	11	24	15	0	20	33	6	49	VTX00354	99%
Div-2	0	0	0	0	0	0	0	0	0	0	0	0	2	VTX00380	98%
Div-3	0	2	0	0	0	2	0	0	0	0	0	0	0	VTX00062	97%
Div-4	93	48	42	0	6	55	17	34	0	66	67	75	17	VTX00054	100%
Div-5	0	0	1	0	0	0	0	0	0	0	0	0	0	VTX00062	97%
Par-1	12	42	14	26	1	16	11	3	0	21	31	7	1	VTX00335	100%
Par-2	0	0	0	0	0	2	0	0	0	0	0	0	0	VTX00350	98%
Arc-1	3	0	0	0	0	0	0	0	0	1	0	0	0	VTX00245	99%
Amb-1	0	2	0	0	0	0	0	0	0	0	2	0	0	VTX00283	99%
Total clones	159	206	170	207	199	182	210	205	202	133	134	191	93		
Total richness	7	12	10	12	15	12	10	7	12	9	9	8	7		

Table S2. Statistical results of the Multiple Response Permutation Procedure (MRPP) testing the pairwise differences in AMF communities between sites.

Site pairs	Delta	*P*
BG / NM	0.52	0.011
BG / ZC	0.45	0.001
BG / GZ	0.56	0.001
BG / GG	0.51	0.001
NM / ZC	0.50	0.001
NM / GZ	0.63	0.001
NM / GG	0.58	0.001
ZC / GZ	0.54	0.001
ZC / GG	0.49	0.001
GZ / GG	0.66	0.029

Table S3. Statistical results of the Multiple Response Permutation Procedure (MRPP) and the analysis of molecular variance (AMOVA) testing the pairwise differences in AMF communities between host plants within a site.

Site	Plant pairs	MRPP		AMOVA	
		Delta	*P*	F	*P*
BG	*S. purpurea* / *L. nanum*	0.47	0.38	1.14	0.31
	S. purpurea / *P. bifurca*	0.47	0.66	1.00	0.45
	L. nanum / *P. bifurca*	0.48	0.92	0.40	0.68
NM	*S. purpurea* / *L. nanum*	0.55	0.05	1.59	0.24
	S. purpurea / *P. bifurca*	0.50	0.37	0.15	0.99
	L. nanum / *P. bifurca*	0.57	0.06	1.56	0.23
ZC	*S. purpurea* / *L. nanum*	0.39	0.19	2.10	0.16
	S. purpurea / *P. bifurca*	0.43	0.34	0.70	0.44
	L. nanum / *P. bifurca*	0.40	0.06	1.99	0.16
GZ	*S. purpurea* / *P. bifurca*	0.72	0.43	1.98	0.17
GG	*S. purpurea* / *L. nanum*	0.59	0.42	0.56	1

Table S4. Physical and chemical characteristics (mean ± SE) of rhizosphere soils collected from different host-site combinations. Data with different lowercase letters and "*" indicate significant levels at $P < 0.05$. " − "indicates no significance. "/" means no data available.

	SOM (g/kg)	N (g/kg)	P (mg/kg)	pH	clay (%)	silt (%)	sand (%)
S. purpurea							
BG	17.9±1.18b	1.21±0.05b	4.74±0.31a	8.48±0.03a	17.55±0.52b	41.72±2.25b	40.73±2.73a
NM	22.15±1.2b	1.28±0.26b	6.04±0.61a	8.9±017b	19.03±0.47b	45.17±2.23b	35.8±2.62a
ZC	19.06±2.1b	1.33±0.3b	5.04±0.17a	8.52±0.04a	15.81±0.71ab	47.1±1.62b	37.1±2.1a
GZ	4.69±1.22a	0.38±0.08a	6.35±0.74a	9.22±0.1c	12.34±1.89a	27.13±5.33a	60.53±7.17b
GG	8.45±1.44a	0.59±0.12a	5.06±0.42a	8.61±0.11ab	15.31±2.01ab	29.49±3.25a	55.2±4.98b
L. nanum							
BG	17.23±1.01b	1.22±0.04b	4.36±0.63a	8.59±0.03a	16.19±0.3a	44.3±1.82a	39.5±2.1a
NM	17.48±0.85b	1.18±0.03b	5.67±0.43a	8.56±0.03a	15.01±0.48a	38.03±1.14a	46.96±1.53a
ZC	17.98±1.46b	1.19±0.11b	4.6±0.36a	8.6±0.02a	14.01±1.35a	42.56±2.87a	43.43±4.09a
GG	6.72±1.36a	0.78±0.19a	5.29±0.51a	9.72±0.08b	21.38±0.65b	42.14±3.34a	36.48±3.97a
P. bifurca							
BG	13.97±3.11b	1.16±0.08a	4.99±0.24a	8.59±0.02ab	17.12±0.72b	43.43±2.51b	39.45±3.2a
NM	21.68±0.99c	1.41±0.29a	4.41±0.55a	8.68±0.05b	18.8±0.42b	48.25±1.95b	32.94±2.01a
ZC	20.02±0.56c	1.32±0.04a	4.32±0.33a	8.54±0.03a	17.69±2.24b	46.4±3.14b	35.9±5.3a
GZ	2.78±0.26a	1.02±0.32a	4.66±0.41a	9.08±0.05c	7.56±2.57a	14.1±5.74a	78.34±8.3b
site	*	*	-	*	*	*	*
plant species	-	-	*	-	-	-	-

Table S5. Statistical results of Mantel test and partial Mantel test examining the correlations between AMF community composition and precipitation, soil variables, plant biomass and coverage.

	mantel		partial mantel	
	r	*P*	*r*	*P*
precipitation	0.54	0.001	0.459	0.001
coverage	0.57	0.00	0.251	0.001
SOM	0.38	0.001	0.071	0.095
pH	0.36	0.001	0.152	0.007
N	0.27	0.001	0.144	0.009
silt	0.17	0.01	-0.064	0.797
sand	0.16	0.013	-0.064	0.851
clay	0.14	0.04	-0.008	0.881
biomass	0.02	0.249		
P	0.02	0.335		

Table S6. Coefficients and *P* values of correlation analyses and partial correlation analyses testing the relationships between either AMF species richness (top) or hyphal length density (bottom) with precipitation, soil variables, plant biomass and coverage.

	Explanatory factor	Correlation analysis		Partial correlation analysis	
		r	*P*	*r*	*P*
AMF species richness	SOM	0.565	0.000	0.318	0.021
	coverage	0.5	0.000	-0.013	0.927
	precipitation	0.411	0.001	0.085	0.547
	pH	-0.401	0.001	-0.153	0.278
	silt	0.309	0.016	-0.045	0.751
	N	0.301	0.018	-0.046	0.746
	sand	-0.274	0.033	-0.045	0.751
	P	-0.219	0.89	/	/
	clay	0.148	0.254	/	/
	biomass	0.13	0.316	/	/
Hyphal length density	precipitation	0.622	0.000	0.332	0.016
	coverage	0.505	0.000	0.019	0.892
	SOM	0.332	0.009	-0.026	0.853
	pH	-0.257	0.046	-0.187	0.185
	N	0.229	0.076	/	/
	biomass	0.194	0.134	/	/
	silt	0.184	0.155	/	/
	sand	-0.177	0.173	/	/
	clay	0.136	0.298	/	/
	P	0.066	0.615	/	/
